# A clustering procedure for three-way RNA sequencing data using data transformations and matrix-variate Gaussian mixture models

**DOI:** 10.1186/s12859-024-05717-6

**Published:** 2024-03-01

**Authors:** Theresa Scharl, Bettina Grün

**Affiliations:** 1 Institute of Statistics, University of Natural Resources and Life Sciences, Vienna, Austria; 2 Institute for Statistics and Mathematics, Vienna University of Economics and Business, Vienna, Austria

**Keywords:** Gaussian mixture, Gene expression, Genomics, Compositional data, Model-based clustering

## Abstract

RNA sequencing of time-course experiments results in three-way count data where the dimensions are the genes, the time points and the biological units. Clustering RNA-seq data allows to extract groups of co-expressed genes over time. After standardisation, the normalised counts of individual genes across time points and biological units have similar properties as compositional data. We propose the following procedure to suitably cluster three-way RNA-seq data: (1) pre-process the RNA-seq data by calculating the normalised expression profiles, (2) transform the data using the additive log ratio transform to map the composition in the *D*-part Aitchison simplex to a $D-1$-dimensional Euclidean vector, (3) cluster the transformed RNA-seq data using matrix-variate Gaussian mixture models and (4) assess the quality of the overall cluster solution and of individual clusters based on cluster separation in the transformed space using density-based silhouette information and on compactness of the cluster in the original space using cluster maps as a suitable visualisation. The proposed procedure is illustrated on RNA-seq data from fission yeast and results are also compared to an analogous two-way approach after flattening out the biological units.

**Supplementary Information:**

The online version contains supplementary material available at 10.1186/s12859-024-05717-6.

## Background

RNA sequencing (RNA-seq) generates huge amounts of information about the transcriptome of the organism studied. In time-course RNA-seq experiments gene expression is observed over time with thousands of genes measured simultaneously using only a small number of samples. The comparison of several experimental conditions induces a three-way data structure. Examples of three-way data are experiments under different process conditions, knock-out experiments or the exploration of different strains.

RNA-seq data provide readouts in form of count data, i.e., read counts per gene. Specific characteristics of the distribution of these counts are non-normality and a dependence of the variance on the mean. The raw count data may be modelled using the Poisson distribution [1] or the Negative-Binomial distribution to account for over-dispersion [2]. Instead of a direct analysis of the raw count data, however, in general several pre-processing steps are applied before analysing the RNA-seq data [3], e.g., to correct for different sequencing depths, library sizes and gene lengths. Typically, log-fold changes of differential expression are calculated and the number of genes is reduced by filtering low normalised counts and low differential expression estimates [4]. For these tasks several R [5] and Bioconductor [6] packages are available, e.g., TCC [7], DESeq2 [8] or edgeR [9].

Time-course gene expression data are analysed in different ways. One possible approach is clustering to find groups of co-expressed genes [10–13]. Different methods and algorithms are used for clustering including *k*-means [14], hierarchical clustering [15], biclustering [16, 17], as well as model-based clustering [18]. Model-based clustering methods embed the clustering problem within a statistical framework and the mixture models used may be adapted in a flexible way to the data structure and clustering aims by specifying suitable models for the components of the mixture.

Using the raw counts, finite mixtures of Poisson as well as Negative-Binomial distributions have been considered for clustering RNA-seq data [19]. Mixtures of multivariate Poisson-lognormal distributions have been proposed for clustering transcriptome sequencing data [1]. Model-based clustering was also extended to three-way data [20] by proposing to use matrix-variate distributions for the components. In this way, the experiments may be assumed to be independent whereas time points are assumed to be dependent. Taking the three-way structure of RNA-seq data under several experimental conditions into account, matrix-variate Poisson-lognormal distributions were used as components [21]. An alternative method to model-based clustering for grouping three-way time-series gene expression data results from extending biclustering to triclustering [22, 23].

Model-based clustering can also be used after applying suitable pre-processing methods to the data. In a two-dimensional setting, a possible approach is to pre-process the RNA-seq data by calculating so-called *normalised expression profiles* and then use data transformations such as the arcsine or logit transformation before clustering the data using Gaussian mixture models [24]. However, both the arcsine transformation as well as the logit transformation have the drawback to be rank deficient and the resulting data are therefore not suitable for model-based clustering with component distributions assuming full rank.

Noting, however, that in fact these normalised expression profiles have similar properties as compositional data [25], one may resort to methods developed for compositional data to analyse the RNA-seq data after normalisation. Compositional data are usually modelled using standard statistical methods after suitably transforming the data with support on the simplex to $\mathbb {R}^D$ or $\mathbb {R}^{D-1}$ where the data transformations are supposed to then facilitate the use of statistical methods based on Gaussian distributions or relying on the Euclidean distance. Aitchison [26] proposed a number of classes of transformed-normal distributions for data on the simplex.

The additive log ratio (ALR) transform is particularly suitable when working with time-course gene expression data as the first time point can be used as a reference. Finite mixture of matrix-variate Gaussian distributions can then be fitted to perform model-based clustering with a suitable model being selected based on a statistical information criterion. A final partition can be obtained by assigning observations to the component with the maximum a-posteriori probability.

For assessing the quality of a clustering several methods have been proposed [27], e.g., the separation between clusters or the compactness of a given cluster. Silhouette width [28] compares the average distance of one observation to members of its own cluster and the average distance to members of its second closest cluster. A modified silhouette width based on posterior probabilities was also developed for model-based unsupervised learning approaches, the density-based silhouette information (dbsi) [29]. The dbsi value of an observation is based on the cluster with the largest posterior probability and the cluster with the second largest posterior probability. In the context of classification, [30] propose a class map to investigate the class-specific performance which takes into account farness from the class as well as the predicted class probabilities. Similarly, we propose in the clustering context a *cluster map* which takes into account cluster separation in the transformed space and compactness in the original space.

In this work we propose the following four-step procedure for clustering three-way RNA-seq data:

Step 1: Pre-processing RNA-seq data where first normalised expression profiles of the genes across time points for a biological unit and experiment are obtained, averages are taken across biological replicates and finally differentially expressed genes are identified to reduce the number of observations.
Step 2: Transforming RNA-seq data using ALR on the normalised expression profiles which have similar properties as compositional data.
Step 3: Model-based clustering of the transformed three-way RNA-seq data using finite mixtures of matrix-variate normal distributions.
  Specify the variance-covariance structure of the components taking into account the experimental design and clustering aims.
  Select the number of components based on the integrated completed likelihood (ICL; [31]) which takes goodness-of-fit as well as cluster separation into account.
Step 4: Post-processing of the cluster solution for validation based on both, the normalised gene profiles as well as the transformed data, and external additional information. Assess the quality of the partition by inspecting the density-based silhouette information (dbsi) based on the posterior probabilities of the individual genes alone as well as in combination with the distance from the cluster center as a measure of compactness in a cluster map. Complement these evaluations with a gene set analysis.

We illustrate this approach on a publicly available fission yeast dataset [32] which has a three-way structure. The data consist of global transcription profiles of two strains, the fission yeast wild type and *atf21* mutant strains, over an osmotic stress time course. The experiment aimed at the identification of genes affected by the knockout of the *atf21* gene. We assess the cluster solution obtained using the proposed procedure also in combination with biological knowledge about functional annotation from the PomBase database [33], the scientific resource for fission yeast. Finally, the proposed three-way clustering approach is compared to a classical two-way approach after flattening out the biological units.

## Material and methods

### Pre-processing RNA-seq data

RNA-seq count data are normalised to account for the varying library size (i.e., the total number of sequenced reads in a sample) and the varying gene lengths [3]. Normalisation of the raw read counts enables the comparison across samples and genes. A comprehensive evaluation of normalisation methods for RNA-seq data is given in [34].

We extend the idea of obtaining *normalised expression profiles* proposed for the two-dimensional data setting [24] to three-way data. The normalised expression profile for gene $i = 1, \ldots , n$, time point $t = 1, \ldots , T$ and experiment $j = 1, \ldots , J$ is given by

$$\begin{aligned} p_{itj} = \frac{x_{itj}/s_{tj} + c}{\sum _{t=1}^T y_{itj}/s_{tj} + c \cdot T}, \end{aligned}$$

with $x_{itj}$ the raw read counts of gene *i* at time point *t* in experiment *j*. These normalised expression profiles are calculated separately for each gene and experiment and give the proportion of reads for gene *i* in experiment *j* with respect to the total reads for gene *i* in experiment *j* across all time points *T* while accounting for the scaling normalisation factors $s_{tj}$ which are time point and experiment specific. A constant *c* is added to avoid potential issues due to zeros. This constant often takes the value 1 but other values, e.g., half of the minimum of all raw counts, are also possible [35]. The scaling normalisation factors $s_{tj}$ can be calculated using, e.g., the DESeq2 normalisation [8].

For each gene profile of a specific gene *i* and experiment *j*, the individual contributions of each time point sum up to one. Therefore, the profile of gene *i* in experiment *j* denoted by $\varvec{p}_{ij} = (p_{itj})_{t=1,\ldots ,T}$ has similar properties as *compositional data*. In the dataset of the empirical illustration and, more generally, in time-course experiments, the relative change to time point T0 in gene expression over time is of main interest, implying that T0 represents a natural reference time point.

### Transforming RNA-seq data

Compositional data are assumed to be made up of the relative parts of a whole with all parts being strictly positive [26]. They follow a vector-space structure on the simplex based on log ratios between the compositional parts rather than the usual Euclidean geometry. The *D*-dimensional simplex is defined as

$$\begin{aligned} S^D:= \bigg \{\varvec{x} = (x_1, \ldots , x_D) \in \mathbb {R}^D | \sum _{i=1}^D x_i = \kappa , x_i > 0 \, \forall i\bigg \}, \end{aligned}$$

with $\kappa$ an arbitrary constant which can be set to 1 without loss of generality. The geometrical structure of compositions is referred to as *Aitchison geometry* [36, 37]. For compositional data, interest lies in the relative proportions of the components measured, absolute quantities and units are irrelevant. Compositional data require that all entries are strictly positive. In case there are components equal to zero in the observed data, one needs to deal with these zeroes – usually viewed as rounded zeroes – and add some small positive constant to ensure this requirement [35].

To model compositional data, one can either use a distribution with support on the simplex, e.g., the Dirichlet distribution, or use *transformed-normal distributions* which map compositional data with support on the simplex to $\mathbb {R}^D$ or $\mathbb {R}^{D-1}$. A number of transformations inducing transformed-normal distributions were proposed for data on the simplex [26]: the additive log ratio transform (also known as logistic normal [38, 39]), the centred log ratio transform, and the isometric log ratio transform.

#### Transformations

The *additive log ratio transform* is given by

$$\begin{aligned} ALR (\varvec{x}) = \left( \ln \left( \frac{x_1}{x_D}\right) , \ldots , \ln \left( \frac{x_{D-1}}{x_D} \right) \right) , \end{aligned}$$

where $x_D$ is an arbitrary component which usually, however, has a specific meaning. ALR leads to a non-orthogonal coordinate system.

The *centerd log ratio transform* is given by

$$\begin{aligned} CLR (\varvec{x}) = \left( \ln \left( \frac{x_1}{m(\varvec{x})}\right) , \ldots , \ln \left( \frac{x_D}{m(\varvec{x})} \right) \right) , \end{aligned}$$

where $m(\varvec{x})$ is the geometric mean of $\varvec{x}$. CLR represents a mapping of $S^D \rightarrow \mathbb {R}^D$, i.e., the resulting matrix is rank deficient implying that the empirical variance-covariance matrix of the data is singular. CLR coefficients cannot directly be associated with an orthogonal coordinate system. Hence, an alternative transformation building on CLR was proposed [26], the *isometric log ratio transform*:

$$\begin{aligned} ILR (\varvec{x}) = CLR (\varvec{x}) \Psi ^{\top }, \end{aligned}$$

where $\Psi$ is an orthonormal basis in the hyperplane. There are infinitely many ways to define such an orthonormal basis system, e.g., the use of pivot coordinates [40]. ILR coordinates represent a mapping of $S^D \rightarrow \mathbb {R}^{D-1}$ and also correspond to an isometry, i.e., all metric concepts on the simplex are maintained.

Applying the ALR and ILR compositional data transformations facilitates the use of statistical methods based on Gaussian distributions or relying on the Euclidean distance on the transformed data. For time-course RNA-seq data the ALR transformation is clearly preferable as differential expression can easily be interpreted relative to T0. By contrast, the interpretation of the ILR coordinates is not straightforward. We thus use the ALR transformation with T0 as reference in our proposed workflow.

#### Illustrating the ALR transformation

**Fig. 1:**
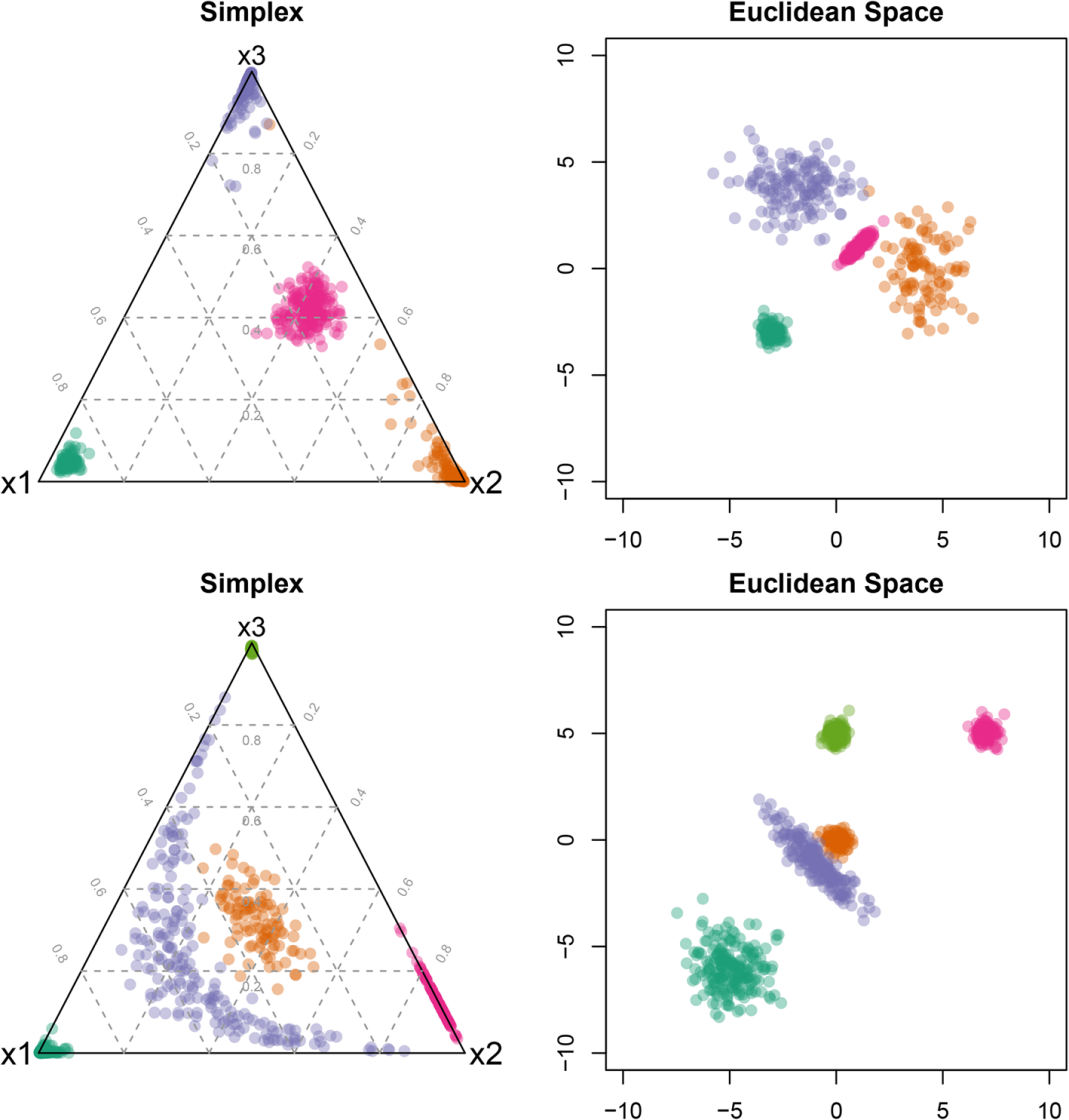
Two examples of artificial data with four (top) and five (bottom) clusters displayed in a ternary diagram (left) and after ALR transformation (right)

In the following we illustrate how clusters on the 3-dimensional simplex transform to Gaussian clusters in 2-dimensional Euclidean space based on the ALR transformation using two different mixture distributions with Gaussian components in Euclidean space. Figure 1 illustrates compositional data in the original space and in the transformed space after applying the ALR transformation. The compositional data take values on the 3-dimensional simplex which are visualised in ternary diagrams on the left. The data are transformed using the ALR transformation and then visualised again using a 2-dimensional scatter plot on the right. The visualised datasets contain either four or five Gaussian clusters in the transformed space. Details about the data generating process can be found in the Additional file 1.

In the first dataset (top row), three clusters are dominated by one component in the compositional vector and therefore these observations are placed in each of the corners of the ternary diagram. The fourth cluster has similar weights for component *x*2 and *x*3 and slightly lower weights for component *x*1 in the compositional vector and therefore its observations are placed approximately in the middle of the ternary diagram. The volume of this pink cluster in the middle is rather comparable to the volumes of the orange and blue clusters on the simplex; however, the volume of this cluster is in comparison much smaller after ALR transformation in the Euclidean space. Also, the pink cluster has a spherical shape on the simplex, but an ellipsoid structure in Euclidean space.

In the second dataset (bottom row), the two greenish clusters in the bottom left and top corner of the simplex differ only slightly in their volumes. In Euclidean space, however, their volumes differ considerably. The orange cluster in the middle has a large volume on the simplex compared to the clusters with observations in the corners or on the edges, while in Euclidean space it is a very compact cluster in the center. This behaviour is even more pronounced for the purple cluster which is spread over a large area of the simplex whereas it has a compact ellipsoid structure in Euclidean space compared to the dark green cluster. In contrast, the orange and light green clusters have the same volume in Euclidean space whereas the light green cluster is much more compact on the simplex.

Figure 1 illustrates that clusters that are very compact in Euclidean space can have a large volume on the simplex. In addition clusters which are dominated by one component might have a larger volume in Euclidean space while being very concentrated in one corner of the simplex. This suggests the need to allow for different volumes and shapes of the clusters in Euclidean space which is not possible using *k*-means and thus requires the use of mixtures of Gaussian distributions where the variance-covariance matrix structure can be specified to allow for different shapes and volumes across clusters as well as allow for dependence between time points and independence between experiments.

### Model-based clustering

The standard form of a *finite mixture model* [41] is

$$\begin{aligned} h(y; \varvec{\Theta }) = \sum _{k=1}^K \pi _k f_k(y;\varvec{\theta }_k), \end{aligned}$$

where *K* is the total number of components, $\pi _k$ is the positive component size of the *k*th component with $\sum _{k=1}^K \pi _k = 1$ and $\varvec{\theta }_k$ is the parameter vector corresponding to the *k*th component distribution $f_k(.;\varvec{\theta }_k)$.

Matrix-variate distributions for the components offer a natural way to model three-way data. This results in a finite mixture model with components distributed as matrix-normal [20]:

$$\begin{aligned} h(\varvec{Y}; \varvec{\Theta }) = \sum _{k=1}^K \pi _k \Phi (\varvec{Y}; \varvec{M}_k, \varvec{\Sigma }_k, \varvec{\Psi }_k), \end{aligned}$$

implying that conditional on component membership, $\varvec{Y} = (y_{jt})_{j=1,\ldots ,J; t=1,\ldots ,\mathcal {T}}$ follows a $J \times \mathcal {T}$-dimensional matrix-normal distribution $(\mathcal{M}\mathcal{N}_{J \times \mathcal {T}})$ with *J* the number of experiments and $\mathcal {T}$ the number of time points. Please note that $\mathcal {T} = T - 1$ is used here to indicate that the number of time points differ between the original space and the transformed space. The parameters of the matrix-normal distribution are the $J \times \mathcal {T}$ mean matrix $\varvec{M}$ and the $J \times J$ and $\mathcal {T} \times \mathcal {T}$ variance-covariance matrices $\varvec{\Sigma }$ and $\varvec{\Psi }$. $\varvec{\Sigma }$ measures the variability along rows (experiments) and $\varvec{\Psi }$ measures the variability along columns (time points). A suitable constraint needs to be imposed on $\varvec{\Sigma }$ and $\varvec{\Psi }$ to ensure identifiability.

The matrix-normal distribution is related to the multivariate normal distribution via

$$\begin{aligned} \varvec{Y} \sim \mathcal{M}\mathcal{N}_{J \times \mathcal {T}}(\varvec{M}, \varvec{\Sigma }, \varvec{\Psi }) \Leftrightarrow \text {vec}(\varvec{Y}) \sim \mathcal {N}_{J\mathcal {T}}(\text {vec}(\varvec{M}), \varvec{\Psi } \otimes \varvec{\Sigma }), \end{aligned}$$

with $\text {vec}()$ the vectorisation operator and $\otimes$ the Kronecker product. Comparing these distributions indicates that much fewer parameters need to be estimated in the case of the matrix-normal distribution (e.g., for $J=2$ and $\mathcal {T}=5$ we get a block matrix of size $10 \times 10$ with $2\cdot 3/2 +5\cdot 6/2=18$ parameters as opposed to $10\cdot 11/2=55$ parameters in a general variance-covariance matrix). As a result of this more parsimonious component specification, more groups of different size, volume and shape can be expected to be found.

The following specifications are imposed on the parameters of the components to obtain suitable cluster solutions. A general matrix with no constraints is assumed for the mean parameter. The variance-covariance matrix measuring variability between the experiments, $\varvec{\Sigma }$, is assumed to be a diagonal matrix as no dependence structure is expected between them, i.e., the experiments are assumed to be independent. Additionally, in order to allow for clusters of different volumes and shapes the values in the diagonal of $\varvec{\Sigma }$ are allowed to vary between dimensions and components. This specification ensures that noise clusters with large volume as well as compact clusters consisting of very similar expression patterns can be identified simultaneously. This represents a major advantage over *k*-means clustering where only global restrictions across all components are possible. Between the time points some correlation structure is assumed. One can either specify a full correlation structure, i.e., $\varvec{\Psi }$ is a general correlation matrix, or restrict $\varvec{\Psi }$ assuming an autoregressive model of order 1 (AR1), resulting in a more parsimonious parameterisation which assumes conditional independence between time points more than one time point apart conditional on intermediate time points [42]. Other component distributions could also be considered, e.g., the *t*-distribution of skewed distributions, to allow for more flexible shapes of the clusters. However, using normal distributions has the advantage that the symmetry and the light tails imply that all observations in the cluster might well be represented by the mean, in particular if the cluster is compact.

To select the number of components, a penalised goodness-of-fit criterion such as the Bayes information criterion (BIC) or the ICL is typically used. These criteria are determined by

$$\begin{aligned} \text {BIC}(K)&= -\ell (.|\hat{\varvec{\theta }}_K) + \frac{\nu _K}{2n}\text {ln}(n),\\ \text {ICL}(K)&= -\ell (.|\hat{\varvec{\theta }}_K) + \frac{\nu _K}{2n}\text {ln}(n) + \text {entropy}, \end{aligned}$$

where $\ell (.|\hat{\varvec{\theta }}_K)$ is the log likelihood evaluated at $\hat{\varvec{\theta }}_K$, which is the maximum likelihood estimate of $\varvec{\theta }_K$, $\nu _K$ is the number of free parameters in the mixture model with *K* components and *n* is the number of genes. The difference between BIC and ICL is the penalty factor, i.e., the entropy which is added for the ICL and which measures the ability of the *K*-component model to provide a well separated partition of the data. A simulation study on artificial data showed that BIC and ICL had an excellent performance for selecting the number of clusters for mixtures of matrix-variate Gaussian distributions [20]. This is in line with the literature indicating that the BIC in general performs well in model-based clustering despite not satisfying the regularity conditions [18]. These criteria may also be used to select among different models or transformations [24, 43].

We use the ICL in our proposed workflow because this criterion aims not only at selecting a solution which provides a good fit to the data, but it also takes cluster separation into account. Hence, the selected solution is supposed to provide better results in a clustering context, in particular given that biologists perform cluster analysis to find groups of co-expressed genes where the functionality and the co-regulation is still unclear. Therefore, rather than identifying the true number of underlying clusters in a dataset, it is of interest to identify small compact groups of genes with similar expression patterns across experiments.

### Post-processing of the cluster solution

The selected mixture model can be used to obtain a partition of the data by assigning each observation to the component where the posterior probability of belonging to the component given the observation is maximum. Different methods have been proposed to assess cluster solutions using internal as well as external criteria.

Silhouette information [28] allows to evaluate the quality of a partition based on a distance metric. The silhouette width is determined as the average distance of one observation $\varvec{y}_i = (y_{itj})_{t = 1, \ldots , \mathcal {T}, j = 1, \ldots J}$ to the other members of its own cluster $a(\varvec{y}_i)$ and the minimum average distance to members of its second closest cluster $b(\varvec{y}_i)$.

$$\begin{aligned} s(\varvec{y}_i):= \frac{b(\varvec{y}_i) - a(\varvec{y}_i)}{\text {max}(a(\varvec{y}_i), b(\varvec{y}_i))} \end{aligned}$$

To avoid the need of specifying a suitable distance metric, an alternative silhouette information was developed based on posterior probabilities which is thus applicable to assess the partition obtained from a mixture model [29]. The density-based silhouette information (dbsi) of $\varvec{y}_i$ is defined as

$$\begin{aligned} \text {dbsi}(\varvec{y}_i) = \frac{\log (\frac{\tau _{k_0}(\varvec{y}_i)}{\tau _{k_1}(\varvec{y}_i)})}{\text {max}_{\iota =1,\ldots ,n} |\log (\frac{\tau _{k_0}(\varvec{y}_\iota )}{\tau _{k_1}(\varvec{y}_\iota )})|}, \end{aligned}$$

where $k_0$ is the cluster with the largest posterior probability $\tau _k(\varvec{y}_i)$ and $k_1$ is the group index for the second largest posterior of observation *i*. In order to avoid numerical instabilities, the posterior probabilities were winsorized to be between 0.00001 and 0.99999 in the numerical implementation. A large value of the dbsi for a given data point is an indicator that this data point firmly belongs to its assigned cluster whereas a small dbsi value indicates that there is some ambiguity in assigning this data point to one cluster. The dbsi metric allows to assess the separation between clusters in the transformed data space.

The *dbsi information plot* visualizes for each gene the dbsi value obtained grouped by cluster, with the values within each cluster being in decreasing order. This plot provides insights into the cluster sizes as well as the separation of the clusters. Based on this plot, one can assess how well the fitted mixture model allows to classify the observations into the clusters. For each cluster, one can also assess how easily which proportion of the genes can be assigned to this cluster.

The dbsi information plot focuses on cluster quality in the transformed space based on the mixture model. We complement this information by combining the dbsi values with a measure of closeness to the cluster center in the original space based on the Euclidean distance. We build on a two-dimensional visualisation method recently proposed for a detailed diagnostic of the quality of a classification procedure where true class memberships are known (class map; [30]). In this diagnostic plot, the probability of the best alternative class is plotted for each observation against the “farness” from the given class.

A similar approach can also be used in an unsupervised setting. To allow for a joint assessment of the solution in the original as well as the transformed space, we propose a *cluster map* which is obtained by plotting the dbsi from the mixture model fitted to the data in the transformed space against the distance to the cluster center in the original space with observations split into different facets based on cluster membership. The distance to the cluster center is determined using a scaled version of the Euclidean distance such that the values are in [0, 1]:

$$\begin{aligned} d_{\varvec{p}_i, c(\varvec{p}_i)} = \frac{\sqrt{\sum _{j=1}^J \sum _{t=1}^T (p_{itj} - c(\varvec{p}_i)_{tj})^2}}{\text {max}_{\iota =1,\ldots ,n} d_{\varvec{p}_\iota , c(\varvec{p}_\iota )}}, \end{aligned}$$

where $\varvec{p}_i$ is observation $\varvec{y}_i$ in the original space and $c(\varvec{p}_i)$ is the transformed estimated mean of the component with the largest posterior. The distance indicates how compact a cluster is in the original space, whereas dbsi allows to assess cluster separation in the transformed space based on the mixture model.

The cluster map plot consists of scatter plots of distance versus dbsi values for each of the clusters in facets. To better identify the regions of points and facilitate comparison across clusters, we suggest to add convex hulls for the observations of each cluster. From a biological point of view we are interested in well separated and compact clusters allowing for easy interpretation of the functionality of the contained genes. This implies that an ideal cluster would be one where observations assigned to the cluster have high dbsi values and a low distance to the cluster center and hence, all observations are located in the top left corner of this scatter plot.

Overall, the dbsi information plot as well as the cluster map scale well in the number of experiments, the number of time points and the number of clusters and are therefore well suited for high-dimensional data containing many observations. Traditional visualisations of the clusters in the transformed and original space can become quite challenging as the dimensionality, especially the number of experiments, increases.

In addition to evaluating cluster solutions based on internal criteria, cluster solutions on genomic data are also typically assessed using external information, e.g., taking into account functional groupings of the genes such as gene ontology (GO) terms. Individual clusters are evaluated regarding gene set enrichment of functional groups. For fission yeast, the gene association file containing GO term information is available from PomBase [33].

### Software implementation

The procedure can be implemented combining several R packages. Package coseq [24, 44] implements calculation of the normalised expression profiles (Step 1). Package robCompositions [25] implements data transformations of compositional data including the ALR and the inverse ALR (Step 2). Package MatTransMix [45] implements the matrix-variate normal mixture model (Step 3), assuming a general variance-covariance matrix $\varvec{\Psi }$. The restricted autoregressive version of order 1 (AR1) is fitted with an implementation of the expectation-maximisation algorithm using code available at the GitHub project page. For fitting the restricted version, the best solution obtained using MatTransMix with the general specification for $\varvec{\Psi }$ is used for initialisation. The restricted version is thus obtained in a subsequent refinement step given the solution for a general variance-covariance matrix $\varvec{\Psi }$. To fit mixture models to a two-dimensional version after flattening out the biological units, packages mclust [46] and Rmixmod [47] can be used for maximum likelihood estimation of finite mixtures of multivariate normal distributions.

Code for the proposed workflow including also the visualisation methods for post-processing the cluster solution (Step 4) is available at the GitHub project page. The repository contains the R code for the newly developed cluster map, the dbsi plot and scripts for reproducing the entire data analysis performed in this study.

## Results

### Analysing the fission yeast dataset

We applied the proposed workflow to a dataset collected in a study where global changes in transcript and protein levels in the fission yeast stress response were investigated [32]. The fission yeast data can be downloaded from the Gene Expression Omnibus and is also available in the Bioconductor package fission. The data comprises global transcription profiles of two strains, the fission yeast wild type (WT) and the *atf21* mutant (Mut) strains, over an osmotic stress time course following treatment with 1 M sorbitol at 0, 15, 30, 60, 120 and 180 minutes. Strand-specific single end sequencing of total RNA was performed in biological triplicates on the Applied Biosystems SOLiD 5500xl Genetic Analyzer System. In total there are $n = 7039$ genes, $T = 6$ time points and $J = 2$ different experimental units for 3 biological replicates. The yeast samples were exposed to oxidative stress, and half of the samples contained a deletion of the gene *atf21* (at locus SPNCRNA.1164). One of the goals of the fission yeast data analysis is to find groups of co-expressed genes over time by additionally taking into account the information about the different experiments.

**Fig. 2:**
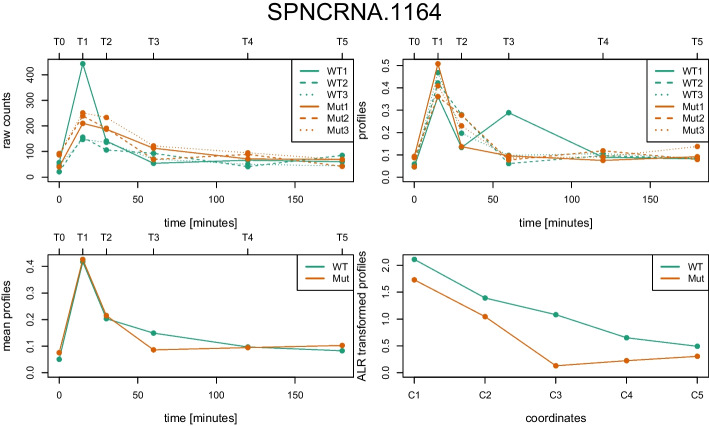
Gene expression profiles of gene *atf21* (at locus SPNCRNA.1164) for the two experiments (WT, Mut) performed in triplicates. Top left: raw counts of six samples taken at 0, 15, 30, 60, 120 and 180 minutes (bottom axis) or alternatively called T0, T1, ...T5 (top axis), top right: normalised expression profiles thereof, bottom left: mean profiles thereof, bottom right: corresponding ALR coordinates

The raw read counts of each of the six experiments were used to calculate the normalised expression profiles using the coseq package. After taking the means over the biological replicates, the normalised expression profiles were transformed to ALR coordinates using the robCompositions package. In Fig. 2, the data associated with each of these pre-processing steps are visualised for the knockout gene *atf21*. The raw counts are shown in the top left panel for all six experiments. In the top right panel the normalised expression profiles are given which sum up to one for each experiment and all values are therefore in the range between zero and one. In the bottom panels the corresponding mean profiles (left) and ALR coordinates (right) are displayed.

A differential expression analysis for gene filtering was performed using DESeq2. Following the DESeq2 workflow [48], a design formula was used that models the strain difference at time point T0 = 0, the difference over time, and any strain-specific differences over time. Differentially expressed genes between the strains at any two time points were selected by taking only genes with an adjusted *p*-value below 0.01 and an absolute log2 fold change larger than 1. These thresholds correspond to the defaults usually employed and yield a subset of 769 differentially expressed genes. Selecting different thresholds would result in a different set of differentially expressed genes. Given that clustering aims at suitably partitioning the set of given objects, clearly a different set would also induce a different cluster solution. In this subset, the most abundant GO terms are GO:0005634 (nucleus, 361 genes), GO:0005829 (cytosol, 284 genes), GO:0005737 (cytoplasm, 147 genes) and GO:0005515 (protein binding, 111 genes).

Model-based clustering of the transformed three-way data was performed using R package MatTransMix. Models with the number of components between 1 and 20 were fitted using a diagonal variance-covariance matrix for the experiments and a full correlation matrix over time. ICL selects the 10 components solution for the ALR transformed data. This is in line with the BIC which would also point to the 10 components solution. These solutions are used to initialise the fitting of the restricted version based on AR1 to account for temporal correlation. For the restricted versions, both ICL and BIC also suggest to use the solution with 10 components given that the criteria show a considerable decrease up to 10 components and are quite flat afterwards. Based on these criteria, these AR1 restricted solutions are preferable to the full versions. In the following, we consider the 10-component solution obtained with the AR1 restriction.

**Fig. 3:**
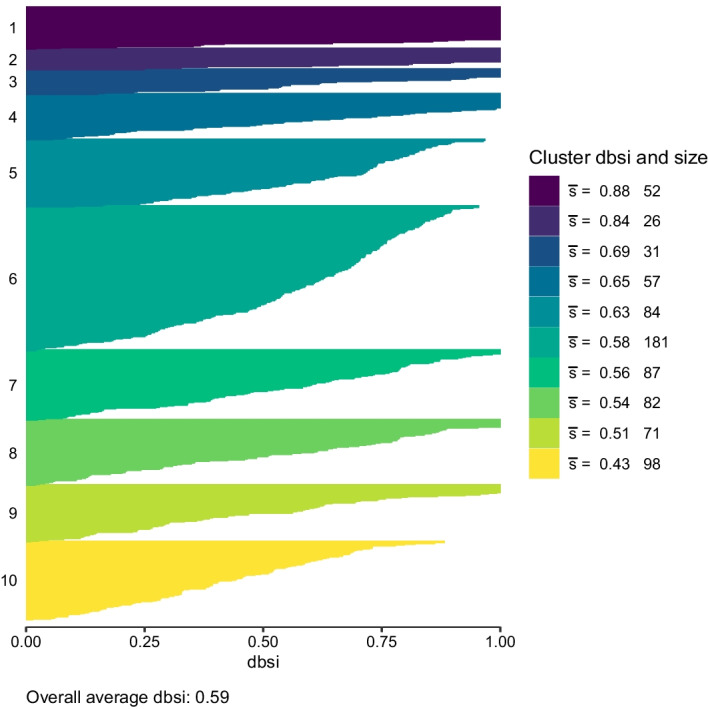
The dbsi information plot of the cluster solution with 10 components

Figure 3 provides the dbsi information plot for the cluster solution obtained, with the clusters sorted by their average silhouette width. The cluster sizes range from 26 to 181 genes indicating there is not a single cluster which would contain the majority of genes. Clearly, in general, clusters with high average dbsi values are those which contain only few genes. Clusters 1 and 2 contain only a rather small number of genes and show a very good separation for all genes contained resulting in high average dbsi values. By contrast, cluster 10 has the smallest average dbsi value and therefore the worst separation. The overall average dbsi is 0.59 which is a moderate value for the dbsi. Results thus indicate that in the transformed space some clusters were identified where the observations can be clearly assigned to these clusters, whereas for other clusters assignment is rather ambiguous.

**Fig. 4:**
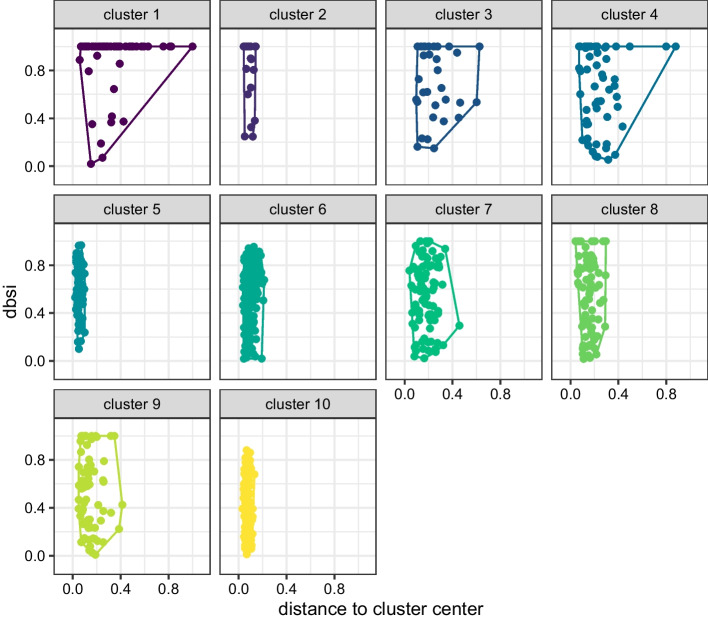
The cluster map plot for the cluster solution with 10 components with facets for each cluster and cluster-specific convex hulls for the observations

In order to get a more detailed view on the quality of this cluster solution, we inspect the cluster map in Fig. 4. For the cluster solution obtained, we can see that even though clusters 1 and 2 have very similar average dbsi values, they differ considerably in the distance to their cluster centers. Cluster 2 is a rather compact cluster as indicated by distance, whereas cluster 1 shows the overall largest average distance of the corresponding genes to their cluster center. Cluster 10 on the other hand has a very small average dbsi value but is a very compact cluster in the original space. The same is true for cluster 5.

**Fig. 5:**
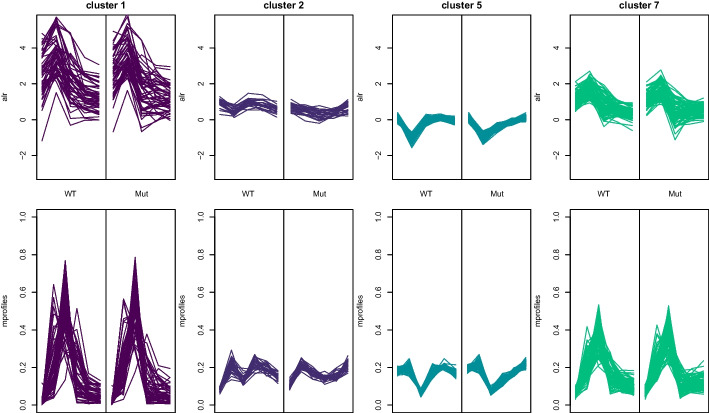
Gene expression profiles of selected clusters with the two experiments shown side by side. Top panel: ALR coordinates in WT and Mut strain, bottom panel: the corresponding normalised expression profiles

We complement the evaluation of the cluster solution and assessment of the quality of specific clusters by also inspecting the gene expression patterns of the individual clusters both in the transformed and original data space. Some selected clusters are visualised in Fig. 5 with the third dimension, i.e., the experiments wild type (WT) and mutant (Mut), shown side by side. This implies that the *x*-axis represents the dimension of time as well as the experiments. Note that such a visualisation is easily possible for this analysis because of the rather low-dimensional nature of the three-dimensional dataset. In the top panel, the ALR transformed data are given for the WT and MuT experiment next to each other. In the bottom panel the corresponding mean profiles in the original space are shown. Cluster 1 which contains genes with good cluster separation but bad compactness is given on the left. As expected, the gene expression profiles vary a lot in their magnitude. Cluster 2 on the other hand is a very compact cluster with good cluster separation. Similar, clusters 5 is again a very compact cluster, whereas cluster 7 is less compact. Cluster 7 also contains gene *atf21* which is shown in Fig. 2. This cluster contains 87 genes which show the highest gene expression 30 min after start of the experiment. The corresponding genes show a very similar expression pattern in the wild type strain (left) and the mutant (right). The composition of the known functionality of these genes is very similar to the global functionality, i.e., GO:0005634 (nucleus, 42 genes), GO:0005829 (cytosol, 46 genes), GO:0005737 (cytoplasm, 14 genes) and GO:0005515 (protein binding, 15 genes).

To highlight the advantages of the three-way clustering approach we also investigate clustering results obtained for the transformed data when using two-way clustering methods as well as *k*-means. For two-way clustering the fission yeast data, we flatten out the third dimension and use a traditional two-way clustering with package mclust. We impose an unconstrained variance-covariance specification to the component distributions because we want to allow for varying volume, orientation and shape across clusters. Flatting out the third dimension, i.e., the experimental conditions, yields a dataset with 769 genes and 10 variables after ALR transformation. Comparing models fitted with 1 to 20 components, a cluster solution with 4 components was selected by ICL (where BIC would have selected 5 components).

**Fig. 6:**
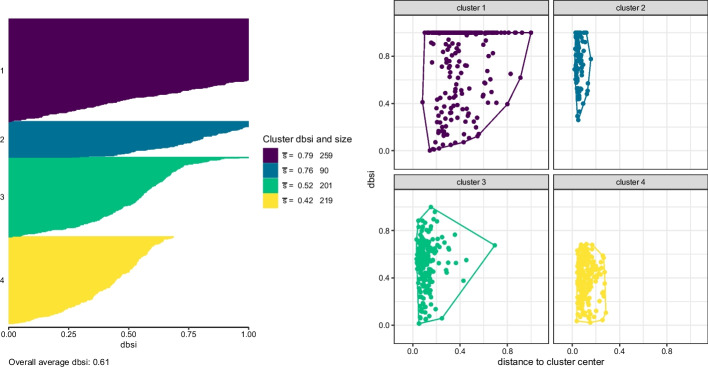
The dbsi information plot (left) and cluster map plot (right) for the two-way cluster solution with 4 components

**Fig. 7:**
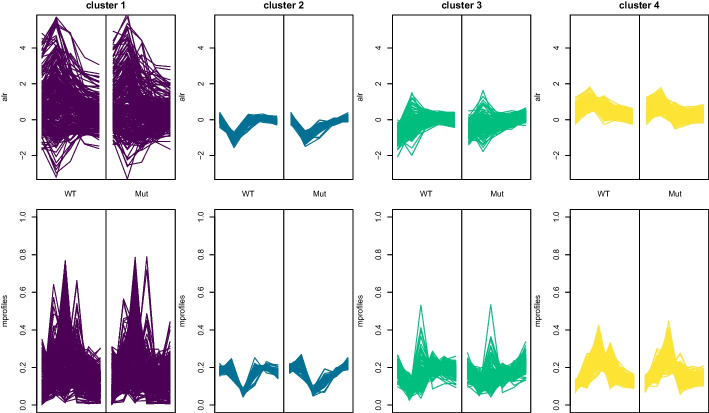
Gene expression profiles of the two-way cluster solution with the two experiments shown side by side. Top panel: ALR coordinates of WT and Mut strain, bottom panel: the corresponding normalised expression profiles

The dbsi information plot as well as the cluster map plot of the two-way clustering solution are given in Fig. 6. The dbsi information plot indicates that in general, the dbsi values are comparable for the two-way and three-way clustering approaches. Cluster 1 is a very large cluster containing a lot of genes with high dbsi values indicating good cluster separation and also some genes with very bad cluster separation. Clusters 2 and 3 are characterised by containing observations where the dbsi values vary considerably. Cluster 4 is generally a cluster with very low dbsi values. The cluster map plot on the right also allows to inspect the compactness of the clusters in the original space. This shows, that in particular cluster 1, which has a good cluster separation according to the dbsi values, contains a lot of genes with large distance to its cluster center. This can also be seen in Fig. 7. Cluster 2 on the other hand is a very compact cluster where the corresponding genes have only a small distance to the cluster center and the gene expression profiles are easily interpretable.

The adjusted Rand index [49] between the three-way cluster solution and the two-way cluster solution is 0.15. This indicates only a rather low congruence between the partitions, most likely due to the difference in number of clusters. The contingency table of the two cluster solutions is given in Table 1 on the left. Good agreement between the cluster solutions is inherent for cluster 9 of the three-way clustering and cluster 4 in the two-way clustering (where only 3 genes were put into different clusters). All genes of cluster 5 in the three-way clustering are contained in cluster 2 of the two-way clustering. However, cluster 2 of the two-way clustering also contains cluster 8 of the three-way clustering and some more genes. This comparison shows that while there is clearly some congruence between several clusters obtained with the two methods there is also a lot of difference between the partitions.

**Table 1:**
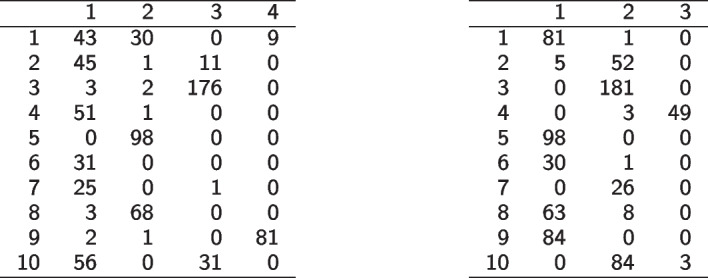
Contingency table of the three-way versus two-way cluster solution (left) and the *k*-means cluster solution (right)

Finally, also the *k*-means algorithm [14] was used for clustering the fission yeast data. The flattend out data to two dimensions was used. Note that this also corresponds to a three-way version of *k*-means which implies isotropic clusters. The number of clusters were selected based on the maximum of the averaged Silhouette width [15] which resulted in three clusters. The comparison of the three-way clustering and the *k*-means clustering solution gives an adjusted Rand index of 0.14 (see Table 1 on the right). In this case an even stronger hierarchical nesting of the clusters of the three-way clustering in the *k*-means partition can be discerned. All observations in clusters 3, 5, 7 and 9 of the three-way clustering are completely contained in one single cluster of the *k*-means solution. For *k*-means clustering, however, no posterior probabilities are available and therefore no dbsi plot and cluster map can be used to investigate the cluster solution in more detail.

The comparison of the three-way clustering solution with the two-way and *k*-means clustering solutions clearly indicates the advantage of using the three-way clustering approach which results in a more parsimonious while still flexible parametrisation of each of the components in the mixture model and hence allows to identify more clusters and thus for a more fine-grained analysis.

### Simulation study

We investigate the performance of the proposed three-way clustering approach in a simulation study using artificial data and compare results to those obtained using two-way clustering and *k*-means clustering. We focus in particular on the ability to determine a suitable number of clusters based on the criteria considered in the application on the fission yeast dataset as well as on the congruence of the partition obtained with the true classification of the observations based on the adjusted Rand index.

We proceed as follows. We generate 100 artificial three-way datasets with the same structure as the fission yeast dataset, i.e., 2 experiments, 5 time points, and 769 genes. As data generating process we use the mixture model estimated to the fission yeast dataset in the three-way clustering procedure, i.e., the 10-component mixture of matrix-variate Gaussian distributions imposing the AR1 restriction on the column-wise covariance matrix. We draw class assignments based on the component sizes and—given class assignment—we draw observations from the matrix-variate Gaussian distribution with mean and variance-covariance matrices estimated for the component.

For each dataset, we use four different methods to cluster these observations: three-way clustering using a full column-wise covariance matrix, three-way clustering using AR1, two-way clustering and *k*-means clustering. For the three model-based approaches we select the suitable number of clusters using the ICL and consider the BIC as well to assess its performance. For *k*-means we use the maximum average Silhouette width as criterion to select the number of clusters as well as fixed the number of clusters to 10, the true number of clusters.

**Fig. 8:**
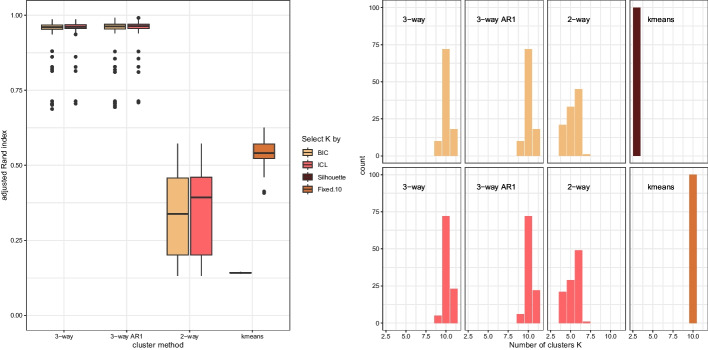
Simulation study on 100 artificial datasets. Left: Boxplots of the adjusted Rand index between the true cluster memberships and the cluster solution. Right: number of selected clusters where the true number of clusters is 10

The adjusted Rand index between the true cluster memberships and the eight different clustering solutions are given on the left of Fig. 8. The results clearly indicate the superiority of the three-way clustering to obtain similar partitions to the true classification than two-way clustering or *k*-means clustering. Three-way clustering using AR1 slightly outperforms classical three-way clustering using a full column-wise covariance matrix. For *k*-means only assuming that the number of clusters is known leads to some reasonable congruence with the true classification, while results are poor if the number of clusters are selected based on the Silhouette width criterion. Regarding the use of either ICL or BIC to select the number of clusters in a model-based clustering context, the simulation study results show that this choice has only a very minor impact on the performance. Figure 8 on the right shows the number of clusters selected by the procedures. Clearly for three-way clustering, the selected number of clusters are quite close to the true number regardless of if the full or the AR1 restricted variants are considered. Two-way clustering and *k*-means select consistently a much lower number of clusters which is also in line with the results in the application on the fission yeast dataset.

The results of the simulation study clearly indicate that the three-way clustering procedure performs well and using the ICL (but also the BIC) for selecting the number of clusters is a reasonable choice. Regarding the use of two-way clustering and *k*-means one might expect to clearly underestimate the true number of clusters present in a dataset.

## Conclusions

In this work we proposed a new workflow for analysing three-way RNA-seq data, i.e., where genes are investigated over time under different experimental conditions. The four-step procedure consists of (1) pre-processing RNA-seq data, (2) transforming RNA-seq data, (3) model-based clustering using matrix-variate component distributions and (4) post-processing the cluster solution obtained. For pre-processing, we propose to calculate normalised expression profiles over time which have similar properties as compositional data. After applying the ALR data transformation, one may assume a matrix-variate normal distribution for the clusters in the data. We thus propose to fit a finite mixture model with components distributed as matrix-normal. A new visualisation method was developed for post-processing of the cluster solution. The cluster map visualises the density-based silhouette information (dbsi) calculated from the posterior probabilities in the transformed space and the distance of a gene to its cluster center in the original space. The proposed workflow was applied to a dataset from fission yeast which has a three-way structure. We also compared results from the three-way clustering with a two-way clustering approach and *k*-means clustering after flattening out the experiments. Results indicate that the three-way approach allows for a more detailed view on the data and encourages the detection of groups of genes with similar temporal expression patterns over time across the different experiments.

The application focused on three-variate data consisting only of two experiments in one dimension. In the future we want to extend our approach to datasets with more than two experiments. In such situations it might be worthwhile to investigate the use of a more parsimonious specification of the mean vectors based on a regression model where the covariates characterise the experimental units. Additionally, it would also be interesting to compare the presented three-way clustering approach to triclustering.

### Supplementary Information

**Additional file 1.** Artificial data used in Fig. 1.

## Data Availability

The dataset used is available in the Bioconductor package fission. R code for the implemented workflow is available at the GitHub project page: https://github.com/theresascharl/RNAseq_clustering

## Abbreviations

RNA-seq: RNA sequencing
ALR: Additive log ratio
BIC: Bayes information criterion
ICL: Integrated completed likelihood
AR1: Autoregressive of order 1
dbsi: Density-based silhouette information
WT: Wild type
CLR: Centered log ratio
ILR: Isometric log ratio
Mut: Mutant
GO: Gene ontology

## References

[1] Silva A, Rothstein SJ, McNicholas PD, Subedi S (2019). A multivariate Poisson-log normal mixture model for clustering transcriptome sequencing data. BMC Bioinform.

[2] Anders S, Huber W (2010). Differential expression analysis for sequence count data. Genome Biol.

[3] Korpelainen E, Tuimala J, Somervuo P, Huss M, Wong G (2014). RNA-seq data analysis: a practical approach.

[4] Bourgon R, Gentleman R, Huber W (2010). Independent filtering increases detection power for high-throughput experiments. Proc Natl Acad Sci USA.

[5] R Core Team (2023). R: A Language and Environment for Statistical Computing.

[6] Huber W, Carey VJ, Gentleman R, Anders S, Carlson M, Carvalho BS, Bravo HC, Davis S, Gatto L, Girke T, Gottardo R, Hahne F, Hansen KD, Irizarry RA, Lawrence M, Love MI, MacDonald J, Obenchain V, Oleś AK, Pagès H, Reyes A, Shannon P, Smyth GK, Tenenbaum D, Waldron L, Morgan M (2015). Orchestrating high-throughput genomic analysis with Bioconductor. Nat Methods.

[7] Sun J, Nishiyama T, Shimizu K, Kadota K (2013). TCC: An R package for comparing tag count data with robust normalization strategies. BMC Bioinform.

[8] Love MI, Huber W, Anders S (2014). Moderated estimation of fold change and dispersion for RNA-seq data with DESeq2. Genome Biol.

[9] Robinson MD, McCarthy DJ, Smyth GK (2010). edgeR: a Bioconductor package for differential expression analysis of digital gene expression data. Bioinformatics.

[10] Androulakis IP, Yang E, Almon RR (2007). Analysis of time-series gene expression data: methods, challenges, and opportunities. Annu Rev Biomed Eng.

[11] Nueda MJ, Tarazona S, Conesa A (2014). Next maSigPro: updating maSigPro Bioconductor package for RNA-seq time series. Bioinformatics.

[12] Scharl T, Voglhuber I, Leisch F (2009). Exploratory and inferential analysis of gene cluster neighborhood graphs. BMC Bioinform.

[13] Srivastava H, Ferrell D, Popescu GV (2022). NetSeekR: a network analysis pipeline for RNA-seq time series data. BMC Bioinform.

[14] Hartigan JA, Wong MA (1979). Algorithm AS136: a $$k$$-means clustering algorithm. Appl Stat.

[15] Kaufman L, Rousseeuw PJ (1990). Finding groups in data.

[16] Hartigan JA (1972). Direct clustering of a data matrix. J Am Stat Assoc.

[17] Pontes B, Giráldez R, Aguilar-Ruiz JS (2015). Biclustering on expression data: a review. J Biomed Inform.

[18] Fraley C, Raftery AE (2002). Model-based clustering, discriminant analysis and density estimation. J Am Stat Assoc.

[19] Si Y, Liu P, Li P, Brutnell TP (2013). Model-based clustering for RNA-seq data. Bioinformatics.

[20] Viroli C (2011). Finite mixtures of matrix normal distributions for classifying three-way data. Stat Comput.

[21] Silva A, Qin X, Rothstein SJ, McNicholas PD, Subedi S (2023). Finite mixtures of matrix variate Poisson-log normal distributions for three-way count data. Bioinformatics.

[22] Amar D, Yekutieli D, Maron-Katz A, Hendler T, Shamir R (2015). A hierarchical Bayesian model for flexible module discovery in three-way time-series data. Bioinformatics.

[23] Jung I, Jo K, Kang H, Ahn H, Yu Y, Kim S (2017). TimesVector: a vectorized clustering approach to the analysis of time series transcriptome data from multiple phenotypes. Bioinformatics.

[24] Rau A, Maugis-Rabusseau C (2018). Transformation and model choice for RNA-seq co-expression analysis. Brief Bioinform.

[25] Filzmoser P, Hron K, Templ M. Applied compositional data analysis: with worked examples in R. Springer series in statistics. Switzerland: Springer; 2018. 10.1007/978-3-319-96422-5.

[26] Aitchison J (1982). The statistical analysis of compositional data. J R Stat Soc Ser B (Methodol).

[27] Hennig C, Hennig C, Meila M, Murtagh F, Rocci R (2015). Clustering strategy and method selection. Handbook of cluster analysis.

[28] Rousseeuw PJ (1987). Silhouettes: A graphical aid to the interpretation and validation of cluster analysis. J Comput Appl Math.

[29] Menardi G (2011). Density-based silhouette diagnostics for clustering methods. Stat Comput.

[30] Raymaekers J, Rousseeuw PJ (2022). Silhouettes and quasi residual plots for neural nets and tree-based classifiers. J Comput Graph Stat.

[31] Biernacki C, Celeux G, Govaert G (2000). Assessing a mixture model for clustering with the integrated completed likelihood. IEEE Trans Pattern Anal Mach Intell.

[32] Leong HS, Dawson K, Wirth C, Li Y, Connolly Y, Smith DL, Wilkinson CRM, Miller CJ (2014). A global non-coding RNA system modulates fission yeast protein levels in response to stress. Nat Commun.

[33] Harris MA, Rutherford KM, Hayles J, Lock A, Bähler J, Oliver SG, Mata J, Wood V (2021). Fission stories: using PomBase to understand *Schizosaccharomyces pombe* biology. Genetics.

[34] Dillies M-A, Rau A, Aubert J, Hennequet-Antier C, Jeanmougin M, Servant N, Keime C, Marot G, Castel D, Estelle J, Guernec G, Jagla B, Jouneau L, Laloë D, Gall CL, Schaëffer B, Crom SL, Guedj M, Jaffrézic F (2013). French StatOmique consortium: a comprehensive evaluation of normalization methods for illumina high-throughput RNA sequencing data analysis. Brief Bioinform.

[35] Pawlowsky-Glahn V, Buccianti A (2011). Compositional data analysis: theory and applications.

[36] Pawlowsky-Glahn V, Egozcue JJ (2001). Geometric approach to statistical analysis on the simplex. Stoch Environ Res Risk Assess.

[37] Egozcue JJ, Pawlowsky-Glahn V, Mateu-Figueras G, Barcelo-Vidal C (2003). Isometric logratio transformations for compositional data analysis. Math Geol.

[38] Blei DM, Lafferty JD (2007). A correlated topic model of Science. Ann Appl Stat.

[39] Russo M, Singer BH, Dunson DB (2022). Multivariate mixed membership modeling: inferring domain-specific risk profiles. Ann Appl Stat.

[40] Fišerová E, Hron K (2011). On the interpretation of orthonormal coordinates for compositional data. Math Geosci.

[41] McLachlan GJ, Peel D (2000). Finite mixture models.

[42] Anderlucci L, Viroli C (2015). Covariance pattern mixture models for the analysis of multivariate heterogeneous longitudinal data. Ann Appl Stat.

[43] Thomas I, Frankhauser P, Biernacki C (2008). The morphology of built-up landscapes in Wallonia (Belgium): a classification using fractal indices. Landsc Urban Plan.

[44] Godichon-Baggioni A, Maugis-Rabusseau C, Rau A (2017). Clustering transformed compositional data using $$k$$-means, with applications in gene expression and bicycle sharing system data. J Appl Stat.

[45] Zhu X, Sarkar S, Melnykov V (2022). MatTransMix: an R package for matrix model-based clustering and parsimonious mixture modeling. J Classif.

[46] Scrucca L, Fop M, Murphy TB, Raftery AE (2016). mclust 5: clustering, classification and density estimation using Gaussian finite mixture models. R J.

[47] Lebret R, Iovleff S, Langrognet F, Biernacki C, Celeux G, Govaert G (2015). Rmixmod: the R package of the model-based unsupervised, supervised, and semi-supervised classification Mixmod library. J Stat Softw.

[48] Love MI, Kim SAV, Huber W (2016). RNA-seq workflow: gene-level exploratory analysis and differential expression [version 2; peer review: 2 approved]. F1000Research.

[49] Hubert L, Arabie P (1985). Comparing partitions. J Classif.

