# Supplementary material for: A clustering procedure for three-way RNA sequencing data using data transformations and matrix-variate Gaussian mixture models
Source: BMC Bioinformatics. 2024 Mar 1;25:90. doi: 10.1186/s12859-024-05717-6 (PMC10905927; doi:10.1186/s12859-024-05717-6)
Supplement: Supplementary file 1 — Additional file 1. Artificial data used in Fig. 1. [file 12859_2024_5717_MOESM1_ESM.pdf]

Supplementary Information to: A Clustering Procedure for Three-Way RNA Sequencing Data Using Data Transformations and Matrix-Variate Gaussian Mixture Models

Theresa Scharl[1] and Bettina Grün[2]

[1] Institute of Statistics, University of Natural Resources and Life Sciences, Vienna

[2] Institute for Statistics and Mathematics, WU Vienna University of Economics and Business

Artificial data used in Figure 1

The data visualised in Figure 1 were generated in the following way.

Dataset 1

The first dataset contains four clusters where each cluster consists of data generated from multivariate Gaussian distributions. In particular, we constructed:

- a cluster of 100 points from $\mathcal{N}\left(\boldsymbol{\mu}_1 = \begin{pmatrix}-3\\-3\end{pmatrix}, \boldsymbol{\Sigma}_1 = \begin{pmatrix}0.1 & 0\\0 & 0.1\end{pmatrix}\right)$,
- a cluster of 100 points from $\mathcal{N}\left(\boldsymbol{\mu}_2 = \begin{pmatrix}4\\0\end{pmatrix}, \boldsymbol{\Sigma}_2 = \begin{pmatrix}1 & 0\\0 & 2\end{pmatrix}\right)$,
- a cluster of 150 points from $\mathcal{N}\left(\boldsymbol{\mu}_3 = \begin{pmatrix}-2\\4\end{pmatrix}, \boldsymbol{\Sigma}_3 = \begin{pmatrix}1.8 & 0\\0 & 1\end{pmatrix}\right)$,
- a cluster of 200 points from $\mathcal{N}\left(\boldsymbol{\mu}_4 = \begin{pmatrix}1\\1\end{pmatrix}, \boldsymbol{\Sigma}_4 = \begin{pmatrix}0.1 & 0.09\\0.09 & 0.1\end{pmatrix}\right)$.

These values were transformed to the simplex using the inverse ALR.

Dataset 2

In the second dataset the five clusters were generated from multivariate Gaussian distributions with

- a cluster of 200 points from $\mathcal{N}\left(\boldsymbol{\mu}_1 = \begin{pmatrix} -5 \\ -6 \end{pmatrix}, \boldsymbol{\Sigma}_1 = \begin{pmatrix} 1 & 0 \\ 0 & 1 \end{pmatrix}\right)$,
- a cluster of 100 points from $\mathcal{N}\left(\boldsymbol{\mu}_2 = \begin{pmatrix} 0 \\ 0 \end{pmatrix}, \boldsymbol{\Sigma}_2 = \begin{pmatrix} 0.1 & 0 \\ 0 & 0.1 \end{pmatrix}\right)$,
- a cluster of 250 points from $\mathcal{N}\left(\boldsymbol{\mu}_3 = \begin{pmatrix} -1 \\ -1 \end{pmatrix}, \boldsymbol{\Sigma}_3 = \begin{pmatrix} 1 & -0.9 \\ -0.9 & 1 \end{pmatrix}\right)$,
- a cluster of 100 points from $\mathcal{N}\left(\boldsymbol{\mu}_4 = \begin{pmatrix} 7 \\ 5 \end{pmatrix}, \boldsymbol{\Sigma}_4 = \begin{pmatrix} 0.1 & 0 \\ 0 & 0.1 \end{pmatrix}\right)$,
- a cluster of 100 points from $\mathcal{N}\left(\boldsymbol{\mu}_5 = \begin{pmatrix} 0 \\ 5 \end{pmatrix}, \boldsymbol{\Sigma}_5 = \begin{pmatrix} 0.1 & 0 \\ 0 & 0.1 \end{pmatrix}\right)$.

These values were transformed to the simplex using the inverse ALR.
